# A Systems Approach for Tumor Pharmacokinetics

**DOI:** 10.1371/journal.pone.0024696

**Published:** 2011-09-14

**Authors:** Greg Michael Thurber, Ralph Weissleder

**Affiliations:** Center for Systems Biology, Massachusetts General Hospital, Harvard Medical School, Boston, Massachusetts, United States of America; Genentech, United States of America

## Abstract

Recent advances in genome inspired target discovery, small molecule screens, development of biological and nanotechnology have led to the introduction of a myriad of new differently sized agents into the clinic. The differences in small and large molecule delivery are becoming increasingly important in combination therapies as well as the use of drugs that modify the physiology of tumors such as anti-angiogenic treatment. The complexity of targeting has led to the development of mathematical models to facilitate understanding, but unfortunately, these studies are often only applicable to a particular molecule, making pharmacokinetic comparisons difficult. Here we develop and describe a framework for categorizing primary pharmacokinetics of drugs in tumors. For modeling purposes, we define drugs not by their mechanism of action but rather their rate-limiting step of delivery. Our simulations account for variations in perfusion, vascularization, interstitial transport, and non-linear local binding and metabolism. Based on a comparison of the fundamental rates determining uptake, drugs were classified into four categories depending on whether uptake is limited by blood flow, extravasation, interstitial diffusion, or local binding and metabolism. Simulations comparing small molecule versus macromolecular drugs show a sharp difference in distribution, which has implications for multi-drug therapies. The tissue-level distribution differs widely in tumors for small molecules versus macromolecular biologic drugs, and this should be considered in the design of agents and treatments. An example using antibodies in mouse xenografts illustrates the different in vivo behavior. This type of transport analysis can be used to aid in model development, experimental data analysis, and imaging and therapeutic agent design.

## Introduction

The pharmacokinetics (PK) of a drug or imaging agent is a major determinant of its utility and efficacy in the clinic. Despite its importance, poor drug distribution and overall tumoral uptake is often neglected as a mechanism of drug resistance in cancer [1] and becomes even more complicated in multidrug regimens [2]. Similarly, low accumulation of imaging agents often reflects poor delivery rather than measurement of the target of interest [3], [4]. The complexity of these issues results in researchers becoming heavily dependent on animal models to test the efficacy of new agents. However, mathematical analysis of the mechanisms involved can provide important insight into the causes of poor uptake and distribution. Given the limited amount of detailed information that can be sampled in animal models and the clinic, these models are finding increasing utility as part of drug and imaging agent development [5], [6].

In this work, we develop a generic model that minimizes the number of suppositions about drug distribution to describe the behavior of therapeutic and diagnostic drugs in tumor environments. We define this ‘systems’ approach as one that does not make any assumptions about which steps are important prior to simulating the uptake, and all the major rates are considered simultaneously. In this manner, the rate limiting step(s) can be unambiguously identified. The purpose of these simulations is not to capture all the highly complex factors affecting drug distribution in tumors but rather to serve as a starting point for identifying the major determinants affecting the distribution of new drugs, to focus more detailed study of pharmacokinetics of specific agents, and to provide a logical, broad overview of the major differences between the distribution of the different class agents. Current pharmacokinetic models are often developed based on static models [7] and from empiric observations based on widely differing assumptions [8], [9]. It is becoming increasingly important to understand the interaction between agents with drastically differing PK profiles, such as with multidrug regimens [10] and in pretargeting strategies [11]. Many of the concepts outlined in this model have been known for some time while others are poorly described in the literature. What is lacking is a broad, self-consistent theory for comparative purposes.

The modeling framework outlined in this work provides a broadly applicable and self-consistent theoretical framework for comparing the uptake of agents in order to better interpret results, design new experiments, and develop more efficacious imaging agents and therapies.

**Figure 1:**
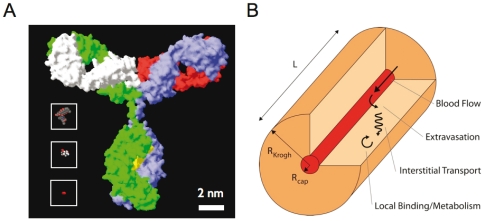
Example molecules and model structure. (A) Space filling models of oxygen, FDG, doxorubicin, and an IgG for size comparison. (B) Diagram of a Krogh cylinder labeled with the four fundamental steps in tumor localization.

We empirically define class I agents as having uptake limited by local tumor blood flow, class II agents having limited vessel permeability and surface area for extravasation, class III agents having limited interstitial diffusion in the tissue, and class IV agents having limited local binding or metabolism of the agent. While the interaction of drug properties and tumor physiology cannot be completely separated, given the large range in drug properties (e.g. 5 orders of magnitude range in permeability), the drug properties dominate the class determination making this analysis useful in drug design. Each of these classes exhibits unique behavior in vivo, and often how an agent is used (systemic versus local delivery, saturating versus subsaturating doses) will affect the class of the agent. To further develop the modeling framework, three instructive examples were analyzed (Figure 1A) as these examples are well studied and span the spectrum of molecular size: oxygen [12], [13], [14], [15], [16], [17], fluorodeoxyglucose [18], [19], and monoclonal antibodies [20], [21].

## Materials and Methods

### Mathematical simulations

Details of the mathematical model can be found in the appendix. Briefly, a Krogh cylinder geometry was used where a cylindrical blood vessel segment is surrounded by tissue with a radius approximately equal to half the local inter-capillary distance (Figure 1B). Blood flows from the arterial end, where a systemic two-compartment model with biexponential decay defines the concentration. The local blood concentration is determined by the blood velocity, permeability of the vessel wall, and fraction of free drug (not bound to blood cells or plasma proteins). Cellular uptake in the blood was ignored (File S1) given the slower kinetics relative to blood flow [22]. A mixed boundary condition is used at the capillary interface, where the flux at the capillary wall determined by the permeability is equal to the diffusive flux into the tissue. In the tissue, the free drug undergoes radial and axial diffusion along with agent specific reaction terms. For small molecules, this involves cellular uptake and metabolism (e.g. oxygen utilization, irreversible trapping over short time scales by FDG phosphorylation, reversible uptake for doxorubicin). For antibodies, this involves reversible binding and dissociation with irreversible internalization. Due to the lack of functional lymphatics in tumors, lymphatic drainage was ignored [23]. The following equations defined the plasma concentration, plasma tissue interface, and tissue concentration (for first order kinetics):
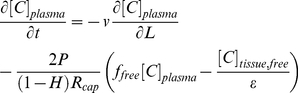
(1)

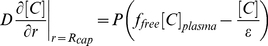
(2)

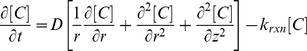
(3)where [C]_plasma_ is the total concentration of drug in the plasma, t is time, v is the local blood velocity, L is the length along the vessel segment, R_cap_ is the capillary radius, H is the hematocrit, P is the vessel wall permeability, f_free_ is the fraction of drug that is unbound, [C]_tissue,free_ is the unbound concentration in the tissue (overall/pseudohomogenous concentration), and epsilon the void fraction. D is the effective diffusion coefficient in tissue, r is the radial distance from a vessel, and k_rxn_ defines the local reaction rate (which is first order in this example equation).

The method of lines was used with axial and radial variations and solved with a stiff solver using Matlab (The Mathworks; Natick, MA). A sparse Jacobian was defined to decrease simulation times.

### Mouse model experiments

For the mouse xenograft experiments, HT-29 and A431 cells were purchased from ATCC (Manassas, VA) and maintained in appropriate media. Antigen expression levels were measured using quantitative beads (Bangs Laboratories; Fishers, IN) per the manufacturer's instructions. For xenograft experiments, 1.5 million cells diluted in PBS were injected subcutaneously in anesthetized nude mice (Cox7 animal facility, Boston, MA). After 2–3 weeks when tumors were approximately 5 mm in diameter, the mice were used for experiments. Cetuximab (ImClone Systems Inc and Bristol-Myers Squibb; Branchburg, NJ) was labeled with VivoTag-680 (Perkin Elmer; Waltham, MA) per the manufacturer's instructions. Either 30 µg or 300 µg was delivered via tail vein 3 days prior to imaging. Mice were imaged using an OV110 small animal imager (Olympus; Center Valley, PA) using appropriate filters for 680 nm wavelength dye. The skin covering the tumors was removed prior to imaging to reduce scattering and variability in depth. The fluorescence intensity of 26 tumors was measured by region of interest analysis using ImageJ (NIH), and statistical analysis was performed in Prism (GraphPad; La Jolla, CA). All animal experiments were carried out in accordance with guidelines from the Massachusetts General Hospital Subcommittee on Research Animal Care (Protocol #2010N000137).

### Pharmacokinetic model

Analysis of the major mechanistic steps of drug delivery in tumors yields the possibility of four major rate-limiting steps (four resistances in series). The first limitation is from local blood flow (Table 1). Molecules in this class are able to easily escape the vasculature and quickly diffuse within the tissue where they are rapidly taken up and/or metabolized [14], [24], [25]. The ability to quickly transport through the tissue actually depletes the concentration along the length of blood vessels, which can be compensated by increasing blood flow. Oxygen and many small molecule drugs (e.g. doxorubicin) fall into this category given their small size and ability to diffuse through membranes. The second limitation is poor extravasation across the vessel walls [20], [26]. While small molecule drugs can often diffuse across membranes, the endothelial cells lining capillaries provide a more formidable barrier to macromolecules and nanoparticles. These large hydrophilic agents cannot easily cross the plasma membrane and access the interstitium by convection and/or diffusion between endothelial cells. Normally, vessels are held together by tight junctions, but these restrictions are slightly relaxed in tumors due to poor vascular formation (e.g. lack of pericyte coverage) and permeability factors (e.g. VEGF) that can also induce fenestrations. However, even with increased permeability relative to normal vessels, this step is often rate limiting for macromolecules such as monoclonal antibodies. The third limitation to drug uptake is interstitial transport. In tumors, elevated interstitial pressure reduces convective transport [27], making diffusion the dominant mechanism of transport (File S1). Extensive reversible tissue binding can further reduce the effective diffusion coefficient, increasing tissue heterogeneity [28]. Many times this is the limiting step for local drug delivery, such as topical delivery or intraperitoneal chemotherapy. Agents that have direct access to the tissue avoid the blood flow and endothelial barriers but often have long diffusion distances within the target tissue [29], [30]. Finally, the last step in localization is the local binding and/or metabolism of the agent. This is often the case for metabolic imaging agents or intracellular enzyme substrates. Here, the final step in localization is rate-limiting, so the total uptake is proportional to the target or cellular process of interest [31].

**Table 1:** Pharmacokinetic Classes.

Class	Uptake Limitation	Examples	Note
I	Blood Flow	Oxygen, doxorubicin,many small molecule drugs	Blood velocity and flow rate is important
II	Extravasation	Antibodies, nanoparticles,many macromolecular drugs	Permeability surface area product(PS/V) is important
III	Diffusion	Oxygen, local drug delivery, highly lipophilic drugs	Spatial distribution of vessels or size of micrometastasis is important
IV	Local Binding/Metabolism	FDG, intracellular protease sensors	Local binding and/or metabolism is important

## Results

### Class Validation

To test the class generalizations, numerical simulations were carried out for sample molecules. The specific parameters used are given in Table 2, and details of these estimates can be found in File S1. The blood supply was fixed for all agents, but the permeability varies by over 5 orders of magnitude. This is caused by the large differences in molecular weight, the ability (or lack thereof) to diffuse across the endothelial cell membrane, and the rate of transport between endothelial cell-cell junctions. Free diffusion coefficients through tissue are also affected by the ability to penetrate cell membranes, the interstitial space, and molecular size. The local binding and metabolism rates depend on the mechanism of local immobilization. For oxygen and doxorubicin, a saturable reaction rate was used which switches from zero order to first order under low concentrations. The binding rate for antibodies assumes that the interaction is high affinity and therefore irreversible [32]. While the rate limiting step for FDG uptake is debated [33], [34], both glut1 transport and hexokinase activity act at the local level. Since FDG competes with endogenous glucose, the uptake rate is first order (File S1). Finally, protease imaging agents are included as a macromolecular class IV agent, and the local step is pinocytosis of the intracellular protease sensor [35].

**Table 2:** Parameters and Groups.

Class	Specific Examples	Blood Flow	Permeability	Diffusion	Reaction
I	Doxorubicin	0.1 mL/g/minL = 500 µm	2.8 µm/s	160 µm^2^/sε = 0.4	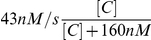
I, III	Oxygen		485 µm/s	1500 µm^2^/sε = 1.0	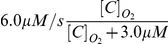
II	Antibodies		0.003 µm/s	10 µm^2^/sε = 0.2	10^5^/M/s[Ag] = 660 nM
IV	FDG		1 µm/s	500 µm^2^/sε = 0.44	5.87×10^−4^/s
IV	Protease Sensors		0.001 µm/s	10 µm^2^/sε = 0.2	1.1×10^−5^/s

* =  dependent on local distance between vessels and plasma concentration.

** =  assuming 1^st^ order cellular uptake.

*** =  assuming a highly expressed target with 10^6^ binding sites per cell.

**Figure 2:**
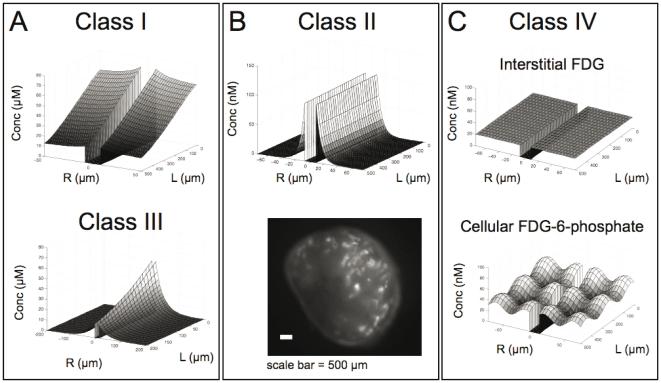
Simulation results for different class agents. (A) Oxygen simulation in region with closely space vessels (50 µm Krogh radius) showing decreasing axial concentration due to poor blood flow (top). With the Krogh radius increased to 200 µm, the radial gradients show diffusion limited uptake for oxygen (bottom). (B) Antibody uptake is heterogeneous due to rapid binding relative to diffusion, and the lack of axial gradients indicates blood flow is not limiting (top). An epifluorescence image of an A431 tumor xenograft slice 24 hrs after 30 µg of cetuximab-VivoTag 680 was injected intravenously shows the perivascular distribution of the antibody (bottom). (C) The blood flow, extravasation, and diffusion are faster than cellular uptake for the class IV agent FDG resulting in homogenous distribution in the interstitium (top). This occurs even with heterogeneous cellular uptake as demonstrated by the intracellular FDG-6-phosphate in the same simulation (bottom).

The simulations for Class I transport show significant axial variation as the molecule is consumed in the tissue and depleted from the blood (Figure 2A, top). These variations indicate that not only is the distance along a vessel and blood flow rate important for oxygen distribution, but also temporal variations have a major effect, a class I trait. Changes in blood flow velocity or temporary stagnation [21], [36] cause transient/acute hypoxia in simulated tissue regions (data not shown). Given the high permeability of oxygen across a single cell layer, this does not present a significant barrier to uptake, consistent with experimental results [16]. In more specialized models, this resistance is often ignored. The heterogeneous vascularization of tumors often creates regions with few to no vessels in the tissue. Using all the same parameters except for the intercapillary distance, a simulation shows a very different behavior in a region with few vessels (Figure 2A, bottom). Here there are significant radial gradients as the oxygen is consumed before it can diffuse to the maximum radius. In this scenario, even if blood flow could be increased without limit, the tissue farthest from the vessel would never be oxygenated due to the diffusive limitation (class III agent). This dependence on the orientation of vessels requires accurate three-dimensional models to recapitulate the in vivo scenario [15], [37].

Class II (e.g. antibodies) display drastically different uptake in tumor tissue, as may be expected given their structure. Simulations for antibody uptake show no detectable axial gradients (Figure 2B, top). These agents are limited by the extravasation rate from the blood, so a minimal amount exits the vessel. In isolated tumor preparations, the miniscule drop in concentration of macromolecules is within the experimental noise [9]. This makes these agents much less susceptible to changes in blood flow, and specialized models will often ignore differences along the length of blood vessels [38]. The capillary wall provides a significant barrier to uptake, so there is a large concentration difference between free drug in the blood and unbound drug in the tissue. This large gradient means extravasation is not highly dependent on the intercapillary spacing as it is for oxygen. What is important is the surface area of active blood vessels (Figure 2B, bottom). This tumor section shows intense perivascular staining of an A431 tumor with a fluorescent antibody except for the region in the lower left. Here a lack of functional blood vessels (no functional surface area) has limited the local uptake. The capillary wall barrier also reduces wash-out from the tumor, resulting in non-specific (size-mediated) enhanced permeability and retention (EPR) effect [39]. The tumor uptake of class II agents from antibodies to nanoparticles can be predicted with this type of modeling [40].

In contrast to the above classes, Class IV agents (e.g. FDG) show uniform tissue concentrations (Figure 2C, top). To ensure this was not an artifact of homogeneous consumption, the uptake rate was arbitrarily given a sinusoidal function in the axial and radial directions. While the internalized FDG-6-phosphate correlated with the uptake rate (Figure 2C, bottom), the interstitial concentration remained constant. This is typical for a class IV agent; the local binding and metabolism rate determines the probe uptake. The localization of this probe is therefore dependent on local glucose consumption, not on delivery. Pugachev et al. showed that FDG colocalized with hypoxic regions that consume more glucose rather than perfusion [41]. This is in contrast to antibodies, where uptake in vitro [42] and in vivo [43] does not correlate to expression unless saturating doses are used.

### Dimensionless Parameters

This systems approach can yield more information than just the rate-limiting step; dimensional analysis of the differential equations is also informative. Given the four major steps in localization, three dimensionless numbers were defined (File S1): the vessel depletion number, Biot number, and Damkohler number. The Biot number and Damkohler number have their origins in differential equations that arise in several fields including heat transfer and chemical reaction and diffusion [44], but the vessel depletion number, being more specific to physiological problems, has only appeared in a few forms (e.g. [45]).

The vessel depletion number is the ratio of extravasation to blood flow rate. A number greater than one indicates the flow rate is important, while a number less than one shows low sensitivity to blood flow. This is defined on both a microscopic scale (velocity along an individual vessel segment) and a macroscopic scale (volume averaged rates). Oxygen has a large ratio, resulting in the decreasing concentration along the axis in Figure 2. This ratio would be much larger were it not for the significant fraction of oxygen bound to hemoglobin in the blood. This bound ‘source’ in the blood helps buffer the loss of oxygen along the length of the vessel, and highly plasma protein bound drugs also show this effect. This occurs for drugs like doxorubicin where 75% of the drug is bound to plasma proteins. FDG has a ratio greater than one, and this affects the distribution at early times after injection discussed below. The vessel depletion number for antibodies is well below unity, and there is no drop in axial concentration.

The Biot number is a ratio of the extravasation rate to diffusion in the tissue and is useful in relating the plasma concentration to the tissue concentration. Oxygen equilibrates rapidly across the endothelium with a Biot number of 5 (with other estimates 30 and higher [46]), in contrast to the concentration of free antibody in the tissue, which is approximately 100-fold lower than what is in the blood. Eventually, bound antibody accumulates to significant levels and this concentration difference is not as apparent. An estimate of the concentration difference across the vascular endothelium during maximum uptake is:
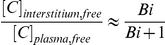



where [C]_interstitium,free_ is the unbound drug in the tissue interstitial space, [C]_plasma,free_ is unbound drug in the plasma, and Bi is the Biot number.

Heterogeneity in the tissue is best captured by the Damkohler number, the ratio of local binding/reaction versus diffusion in the tissue. If the ratio is much less than one, such as with FDG (and oxygen in well vascularized areas), diffusion is faster than immobilization, and there are few radial gradients in the tissue. Ratios much larger than one result in rapid immobilization and large radial gradients, such as with doxorubicin and antibodies. It is important to note that this heterogeneity is caused by the rapid binding rate relative to diffusion (often termed the ‘binding site barrier’ for antibodies [47]), but the rate-limiting step for tumor uptake is still extravasation for antibodies since they are a class II agent.

### Class Variation

Tumor pharmacokinetics are highly complex, and there are notable exceptions to the categorical behaviors presented above. For example, oxygen has been shown to be limited by blood flow in acute hypoxia scenarios but by diffusion in chronic hypoxia. A simpler case is in healthy tissue, where oxygen is not limited by any of these steps but rather tissue metabolism (class IV). The body strives to maintain the supply by redirecting flow and maintaining evenly spaced vessels. To increase oxygen uptake in these tissues, the tissue must simply metabolize (utilize) more oxygen. Oxygen, and other drugs, can therefore vary depending on spatial differences (e.g. local vascularization), temporal changes (e.g. blood flow), and tissue type (healthy versus tumor). Despite these inherent complexities, this systems analysis provides a logical framework in which to discuss and understand these variations. A few informative examples are presented below.

**Figure 3:**
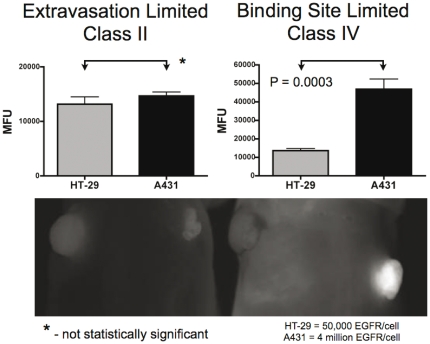
Class variation with antibodies. Mice with HT-29 tumor on the left side and A431 tumors on the right side were injected with 30 µg (left) or 300 µg (right) of cetuximab-VT680. The lower dose is subsaturating, so uptake is limited by delivery from the vasculature with similar uptake in both tumors. At saturating doses, the uptake is limited by the number of binding sites, and uptake is statistically higher in the A431 xenografts, which express EGFR at a much higher level. The reported p values are from a two-tailed t-test.

Antibodies are excellent targeting agents with high binding affinity and specificity, and they are used in the clinic for both therapy and imaging. As macromolecules, they are class II agents, limited by permeability and blood vessel surface area in the tumor. However, with increasing doses, eventually all the binding sites within the tumor are targeted, and the tumor sites become saturated. At this point, further uptake is limited by the lack of free binding sites, not delivery by extravasation. Once saturation has been reached, the antibody behaves as a binding site limited, or class IV, agent. This has major implications for antibodies as imaging agents, since a difference in expression can only be detected at saturating doses. Several groups have demonstrated this non-linear behavior [48] and requirement of saturation [3], [4]. For a direct example, Figure 3 shows a mouse with HT-29 tumors (left) expressing 5x10^4^ EGFR/cell and A431 tumors (right) expressing 4x10^6^ EGFR/cell. Both tumors have similar levels of vascularization, so a subsaturating 30 µg dose results in similar uptake between both tumors. The mouse on the right has the same tumors but was injected with 300 µg of cetuximab, more than enough to saturate the HT-29 tumor. Here there is a statistically significant difference between the tumors. The background also increased given the 10-fold higher dose. This is an important example where an agent switches from class II behavior to class IV behavior based on the dose.

**Figure 4:**
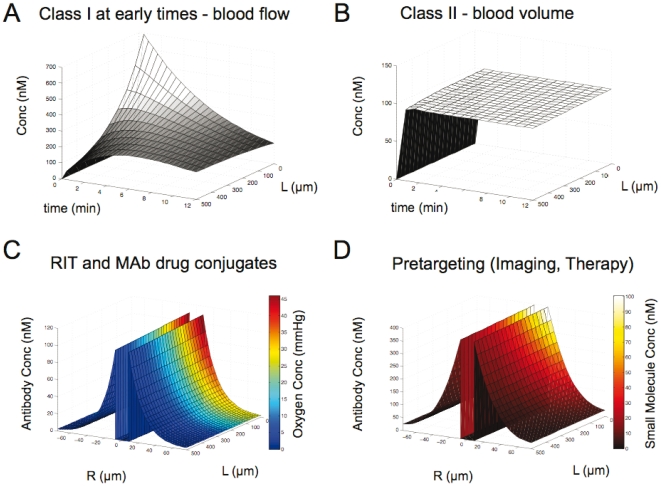
Class variation in time and multi-agent simulations. The plasma profile along the length of the vessel is shown for the first 12 minutes for FDG (A) and an antibody (B). The axial gradients indicate a transient blood flow limitation (class I) for FDG while the antibody evenly fills the blood volume. (C) A joint simulation of oxygen (color scale) and a monoclonal antibody (z-axis) show differential uptake. The antibody is delivered to regions not reached by the blood flow limited oxygen, and other regions are well oxygenated with no antibody. (D) Similarly, a pretargeting simulation with higher antibody dose (z-axis) and reacting secondary agent (color scale) shows some regions targeted by the primary antibody may be missed by the rapidly cleared and blood flow limited small molecule secondary agent.

Variations in distribution can also occur during the initial transient phases of drug distribution. The FDG simulation results in Figure 2C were shown at 1 hr after injection. During the first few minutes after i.v. administration, the drug has not ‘filled up’ the interstitial space in the tumor, and the metabolic uptake does not dominate the distribution. Simulation results for the first 12 minutes after FDG administration are shown in Figure 4A. The concentration along the length of the blood vessel shows steep axial gradients at early times, and at this point, uptake is limited by blood flow. Only after the drug has evenly distributed in the tissue does the heterogeneity vanish. In fact, Mullani et al. have taken advantage of this transient class I behavior by using the FDG signal during the first 2 minutes of administration to measure tumor blood flow [24]. This is in contrast to antibodies (Figure 4B), which show no axial gradients and uniform plasma concentration. Experimentally, this is demonstrated with fluorescent vascular imaging agents [35], which are often macromolecules. The concept of small molecules as blood flow indicators and macromolecules as indicators of blood volume is well known in the MRI community [49].

Although drugs can vary between classes depending on factors such as time, dose, location, and tissue, the drastically varying pharmacokinetic parameters often result in drugs lying well within a particular region (File S1). Even with spatial and temporal variability, drug pharmacokinetics are often dominated by a single class per drug.

### Multidrug regimen

An unbiased approach is beneficial when studying new agents where the rate limiting steps and distribution are currently unknown. However, it is also important for well-studied drugs when used in multi-drug regimens [6]. In these cases, the agents often fall into different classes, and the overlap in concentration between the two drugs is highly variable. For example, oxygen is a class I agent, being limited by blood flow (with class III diffusion limitations near necrotic regions). Hypoxic regions are known to be resistant to radiation and certain types of chemotherapy. Antibodies, class II agents, are currently being explored as carriers for radioisotopes and chemotherapy drugs [50]. A full tumor pharmacokinetic systems analysis shows that these drugs will deliver radiation and chemotherapy to a range in oxygenation levels in the tumor (Figure 4C). Similarly, pretargeting strategies for imaging [51] and therapy [11], [52] typically pair a slowly cleared antibody (class II) with a rapidly cleared small molecule (class I) secondary agent. Colocalization and binding of these agents may only occur in regions with sufficient blood flow (Figure 4D), while antibody in other regions may never be exposed to secondary agent.

Besides illumination of the challenges, the described approach can be used to provide guidance for circumventing a given problem. Judicial use of anti-angiogenic therapies may benefit certain situations. Therapies such as bevacizumab and other anti-angiogenic treatments ‘normalize’ tumor blood vessels by reducing permeability, increasing pericytes coverage, and restoring pressure gradients [53]. A disruption in this pathological signaling pathway may also restore homeostatic mechanisms to increase blood flow [14]. Based on this systems analysis, this results in contrasting effects. Class I agents will have synergistic effects with anti-angiogenic drugs, since this will increase blood flow and delivery into the tissue [54]. In fact, this is how these drugs are often used in the clinic [10]. Class II agents, however, are limited by permeability and will be antagonized by the drug since it is predicted to decrease their uptake. We speculate that this may have played a role in the failure of combining antibody treatments in two recent clinical trials [55], [56], but more investigation into this complex scenario is required.

### Discussion

Here we define four fundamental classes of agents for drug distribution in tumors: those limited by blood flow, vessel permeability, interstitial diffusion, and local binding and metabolism. Identifying the rate-limiting step is important in understanding restrictions on total uptake, developing more complex and specialized pharmacokinetic models, pairing pharmacokinetic models with pharmacodynamic studies (e.g. PK/PD modeling), interpreting experimental results, predicting drug overlap and interactions for multi-drug regimens, and designing novel molecules for imaging and therapy.

### Pharmacokinetic Classes

Blood flow limited agents form the first class of molecules, and this includes oxygen and many small molecule chemotherapeutics such as doxorubicin. Pharmaceutical developers may select for these types of agents when developing orally available drugs. Agents that are able to passively cross the intestinal lining in the gut will also be able to passively cross the endothelium in blood vessels. Biologically, these membranes are very different, and considerations of transporters and specific interactions require more specialized models. However, at the fundamental physiochemical level, both consist of lipid bilayers and aqueous solutions, and drugs that quickly and passively cross the intestinal epithelium may also quickly cross the tumor endothelium.

Class II agents are primarily limited by their permeability across the endothelium and include biologicals (antibodies) and nanomaterials. Because these agents have difficulty crossing membranes, they are often delivered parenterally, such as by intravenous infusion or subcutaneous injection. The slow exit from the vessels does not deplete the concentration even with poor blood flow, so these agents have the potential to access regions of the tumor with low blood flow.

The mechanistic causes of diffusion-limited uptake are likely the most poorly characterized in the literature. While several papers have described diffusion-limited results [9], [57], the reasons why these differ from other similar sized agents has not been well described. This most often occurs for highly lipophilic agents of low molecular weight. Interestingly, their extravasation rates and diffusion rates are typically higher than molecules of similar molecular weight [58]. Given that these molecules have higher permeability and diffusion coefficients than similar but more hydrophilic class I molecules, why are they limited by diffusion? These model results indicate it is due to their high level of plasma protein binding that spares these agents from blood flow limitations. The large fraction of protein bound drug serves as a depot in the blood that maintains the concentration of free plasma drug along the length of the blood vessels. The high lipophilicity allows significant extravasation of free drug, resulting in a class III agent. Since these drugs penetrate cell membranes, the endothelium is just one of many cell layers the drug must pass. This is in contrast to macromolecular class II agents, where the large flat endothelial cells connected by tight junctions form a significant barrier.

Class IV agents are limited in their uptake by local binding and metabolism. Of all the classes, this one is the most difficult to predict. The localization rate is dependent on the specific mechanism of immobilization in the tissue including binding, cellular uptake, metabolism, pinocytosis, or enzymatic activation. Because of this, it is more difficult to generalize the properties of these agents, and it includes small molecules such as FDG and macromolecules like protease sensors [35]. Even more complicated are cases where the result is dose dependent, such as saturating doses of antibodies and extreme cases of necrosis, where even FDG can be limited in uptake [59]. However, this class is very important for developing quantitative imaging agents. Imaging modalities such as PET, SPECT, and MRI are quantitative in nature, but the localization of contrast agents is often not. Many times the localization is dependent on blood flow and/or permeability, not exclusively on the level of target that is being measured. This remains a challenge in the field, and predictive models are very useful in guiding agent design to ensure their uptake correlates with target levels before engaging in time consuming and more expensive in vivo validation.

### Tumor Heterogeneity

Tumor physiology is extremely complex and heterogeneous, and the values of blood flow [21], [25], vascularization [60], vessel distribution [61], and target concentration [43], [62] vary significantly within and between tumors. While drug distribution in tumors is the result of both tumor specific and drug specific parameters, the large range in drug properties dominate the classification. For example, vessel permeability of macromolecules may vary 10-fold between tumors and normal tissue [26], but the permeability of oxygen relative to macromolecules spans 5 orders of magnitude. Only when a drug is borderline between two classes will tumor heterogeneity have a major impact. For example, an antibody close to a saturating dose will saturate more highly vascularized regions prior to less vascularized regions (borderline between class II and class IV). Tumor heterogeneity has a greater influence on the distribution within a particle class. For example, the variability in blood flow to different regions of the tumor will affect the distribution of class I agents, while the variability in vessel surface area will cause heterogeneous distribution of class II agents (e.g. Figure 2b). This heterogeneity between tumor regions is many times correlated; highly vascularized regions often have high blood flow rates, a large vessel surface area, and small diffusion distances between vessels. The opposite is true for semi-necrotic and necrotic regions.

The different agent properties can be used to rationally design probes for measuring these variable tumor parameters and any pharmacologically induced changes. Small tracers with minimal plasma protein binding (class I) will distribute in regions of high tumor blood flow, while macromolecules (class II) will initially fill the blood volume but can be used to measure macromolecular permeability [49].

### Dimensional Analysis

Unbiased analysis of the transport rates also provides insight into the distribution of agents in the tumor. Dimensional analysis results in three numbers that describe the distribution: the vessel depletion number, Biot number, and Damkohler number. The first provides insight into the importance of blood flow in active vessels including the impact of transient and variable tumor blood flow. The Biot number describes the tissue concentration relative to the plasma concentration, often useful for *in vitro/in vivo* correlations. Values much greater than unity indicate equilibrium is achieved, and the plasma concentration is equal to the tissue concentration, at least in close proximity to the vessels. However, for class II agents, this value is often much less than one, and the free drug concentration just outside the vessel is much less than that in the blood. Finally, the Damkohler number describes the rate of immobilization (reaction) to the rate of transport (diffusion). A value much greater than one results in very heterogeneous uptake.

### Pharmacokinetic Simulations

The described analysis of transport may be particularly useful when developing more specialized and sophisticated pharmacokinetic models for specific agents. The tumor microenvironment is extremely complex biologically with heterogeneous physiology. Many of the parameters used in this analysis vary significantly spatially and temporally in tumors. However, the drug uptake and distribution is generally not sensitive to all of these variations and typically is heavily dependent on only a few. This provides an opportunity for model reduction, where several aspects of transport can be ignored since the drug uptake has little or no sensitivity to these parameters. For example, models of antibody transport often ignore issues of blood flow and the corresponding axial variations, since the concentration is not depleted along the length of vessels [38], [63]. Even greater model reduction is possible for FDG. Given the lack of radial and axial gradients, compartmental models have been successful in describing and analyzing FDG data, most notably Patlak analysis [18]. This analysis is also useful for selecting the type of physiologically based pharmacokinetic (PBPK) model for data fitting. In these models, each organ is treated as a separate compartment, but given the large number of parameters, most often the individual rates are fit from experimental data. The systems analysis describes whether transport rates are related to blood flow [8] or extravasation [64]. The collection of rates now available in the literature (File S1) should facilitate the development of more predictive models [65].

Another aspect that enables simplification in Patlak analysis is the assumption of irreversible uptake. This allows the analysis to focus simply on uptake, which is correlated with glucose consumption. While a detailed description of different mechanisms of clearance is beyond the scope of this paper, we will mention them briefly. The first mechanism of clearance results from systemic clearance of the drug, often by the liver (metabolism, biliary excretion) and/or kidney filtration [8]. This reduces the driving force for uptake and can eventually remove drug from the tissues if the gradient is reversed. The second mechanism is local clearance, such as by metabolism in the tissue. A typical example would be the internalization and degradation of antibodies, which have very slow plasma clearance [66], [67]. A final mechanism of clearance is radioactive decay, which is often important with imaging agents. Imaging instrumentation often reports decay-corrected radioactivity, but the activity must be strong enough for sufficient signal to noise ratios and reasonable image capture times. Quickly decaying isotopes such as F-18 require agents that localize on the same time scale before the radioactivity is ‘cleared.’ Identifying both the rate-limiting step in uptake (class I through IV) and major mechanism of clearance is informative, since it is this ratio that determines the maximum uptake in the tumor and the specific time course of drug concentration.

### Conclusions

The factors controlling delivery are extremely complex, and a theoretical analysis is required to try and parse out logical principles. With further development of predictive models, the distribution of drugs will be able to be included in the drug development process for more efficacious therapies. This is very important for small molecule drugs, which are difficult to track with autoradiography. Most small molecule drugs are not characterized at this level, but doxorubicin provides an exception due to its intrinsic fluorescence. By using a systems approach to analyze the pharmacokinetics for imaging agent development, newer agents can be rationally designed for the purpose at hand, such as measuring target expression. In silico design paired with experiments will be an increasingly powerful approach to developing new agents.

Theoretical analysis of tumor pharmacokinetics can provide guiding principles for drug and imaging agent design. Often times experimental results do not agree with the expected outcome, and a variety of factors are discussed that could be the cause of this discrepancy. Many times these deviations are driven by only one or a few of these factors, and this model framework provides the basis for narrowing down the list of possibilities in determining these controlling factors. These principles will become even more important as drug regimens and imaging techniques increase in complexity. This approach can be used by experimentalists to qualitatively understand the different delivery issues or quantitatively estimate the dimensionless parameters that predict uptake and distribution. The parameters outlined here and in the references can be used as a starting point for developing more sophisticated simulations for the molecule of interest. In this manner, a systems approach to tumor pharmacokinetics provides insight into the difficult to measure tumor distribution of drugs and can help in interpreting experimental data, forming more sophisticated pharmacokinetic models, and designing newer, more efficacious imaging agents, drugs, and treatment regimens.

## Supporting Information

File S1 **Dimensional Analysis with 3D Plot, Model Equations, Parameterization, and Additional Validation.** The model equations are used to derive the dimensionless numbers, and the four classes are mapped in three-dimensional space relative to these three groups. References are provided for the parameter values used in the model, and additional simulation results for oxygen and doxorubicin are presented for further model validation.
(DOC)
