# Supplementary material for: A Systems Approach for Tumor Pharmacokinetics
Source: PLoS One. 2011 Sep 14;6(9):e24696. doi: 10.1371/journal.pone.0024696 (PMC3173470; doi:10.1371/journal.pone.0024696)
Supplement: File S1 — Dimensional Analysis with 3D Plot, Model Equations, Parameterization, and Additional Validation. The model equations are used to derive the dimensionless numbers, and the four classes are mapped in three-dimensional space relative to these three groups. References are provided for the parameter values used in the model, and additional simulation results for oxygen and doxorubicin are presented for further model validation. (DOC) [file pone.0024696.s001.doc]

**Supporting Information**

**Outline**

Class Three-Dimensional Diagram

Model equations

Derivation of dimensionless groups

Cellular Uptake

Parameterization

Table of Parameter References

Oxygen

FDG

Doxorubicin

Antibodies and Protease Sensors

Additional Model Validation

Oxygen

Doxorubicin

**Class Three-Dimensional Diagram**

The three dimensionless numbers define the relationship between the four fundamental rates that determine tumor uptake and distribution. The relative values of these numbers map out the four regions for each class of molecule and can be visualized on a single three-dimensional plot.


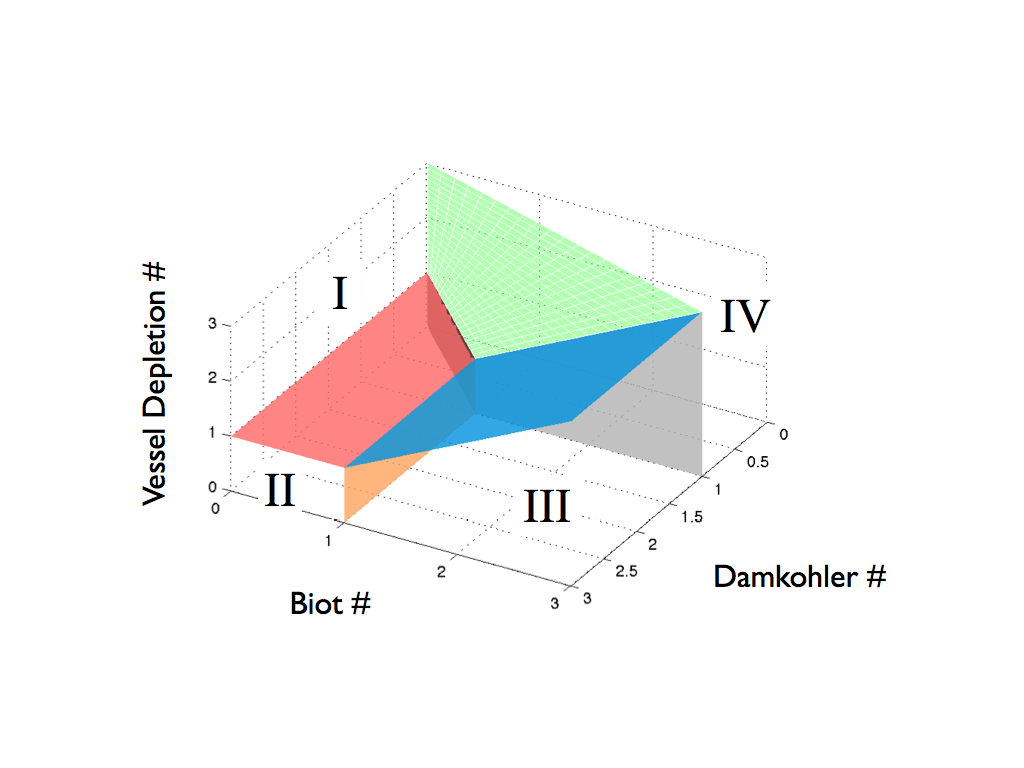


Supplemental Figure 1 – Three-dimensional Class Diagram. The boundaries between the four classes were plotted with the three dimensionless groups along the axes. The colored boundaries divide regions with different rate limiting steps in uptake (Class I = blood flow, Class II = extravasation, Class III = diffusion, and Class IV = local binding/metabolism)

Each of the surfaces above corresponds to relationship between the dimensionless numbers defined below. However, given the spatial and temporal heterogeneity in tumors, these boundaries do not exist as absolute cut-offs but rather transitional regions.

Supplemental Table 1

| **Surface Color** | **Class Division** | **Plane** | **Ratio** |
| --- | --- | --- | --- |
| Red | I/II | VD# = 1 |  |
| Orange | II/III | Bi = 1 |  |
| Light Grey | III/IV | Da = 1 |  |
| Blue | I/III |  |  |
| Dark Grey (obscured) | II/IV |  |  |
| Green | I/IV |  |  |

Note: All surfaces are planar except the green Class I/IV boundary.

**Model equations**

Assumptions

- Axial diffusion in the blood vessel is neglected (relative to convection)

- There are no radial gradients in the plasma

- Diffusion occurs through pseudo-homogenous tissue with an effective diffusion coefficient

- Transport in the tissue is dominated by diffusion

Given the elevated interstitial pressure in tumors[1], convection in the interstitium does not significantly affect drug distribution. This can be captured with an interstitial Peclet number:

where v_i_ = interstitial velocity, R_Krogh_ = spatial length of interest, and D = diffusion coefficient. Convection is highest near the periphery where the interstitial pressure gradients are largest. Even with a velocity of 0.17 μm/s near the periphery, a Krogh radius of 100 μm, and a macromolecular diffusion coefficient of 14 μm^2^/s, the Peclet number is still only order 1. This lack of impact of convection has been numerically simulated to confirm these scaling results[2]. For small molecules with larger diffusion coefficients, convection is even less important.

Capillary concentration

where [C]_plasma_ is the total concentration of drug in the plasma and assuming a rapid equilibrium exists between free and bound drug. R_cap_ is the capillary radius, L is the length along the vessel, H is the hematocrit, v is the blood velocity, P is the vessel wall permeability, f_free_ is the fraction of drug that is unbound, [C]_tissue,free_ is the unbound concentration in the tissue (overall/pseudohomogenous concentration), and epsilon the void fraction. It is assumed there are no significant radial gradients within the vessel.

Simplifying:

Tissue concentration

Assuming only axial and radial gradients:

A universal reaction term cannot concisely be defined for all drugs (see cellular uptake section). For the current simulations:

for oxygen

for FDG

for antibodies and

for free target

for bound target-antibody complex

Boundary conditions

*Tissue*

No flux out of Krogh cylinder

Robin boundary condition at capillary where C is time dependent

No flux out of tissue ends (all enters or leaves from vasculature)

*Capillary*

Entrance is systemic plasma concentration

Using Danckwerts BC at exit

and

**Derivation of dimensionless groups**

Vessel Depletion Number

Non-dimensionalize capillary concentration

where

Defining the vessel depletion number:

So

The blood flow number is defined based on a ‘microscopic’ perspective, where the velocity and length of a single vessel segment are in the definition. This can also be expressed from a ‘macroscopic’ perspective by some additional manipulation:

where the top rate is simply the permeability surface area product and the bottom rate is the flow rate in units of volume of blood per volume of tissue per time (e.g. mL/cm^3^/s).

The macroscopic number does not completely define the microscopic system, since Q is a single parameter to describe blood flow, but the microscopic description contains velocity and length.

Biot Number

Only the free drug is considered to cross the capillary wall. While protein bound drugs (e.g. albumin) can cross the capillary wall, this rate is much slower than free drug. If the permeability for a small molecule is 3 μm/s and the macromolecule is 0.003 μm/s, then 99.9% of the drug would have to be bound to plasma before this rate equals that of the free drug. At such high plasma binding levels, drugs may have trouble exerting their mechanism of action[3].

Non-dimensionalize

To scale the concentration, the free tissue concentration will be used, evaluated at the capillary wall (highest tissue concentration).

where

The Biot number represents the equilibration of free drug across the plasma membrane. With a significant part bound in the blood, the free drug can still equilibrate quickly.

Since the derivative and scaled concentration are approximately equal to one:

As the Biot number becomes very large, the interstitial free drug concentration in the tissue approaches the free drug concentration in the plasma.

Damkohler Number

The relevant local binding and metabolism rate is defined as the first effectively irreversible immobilization step. For example, rapid, linear, and *reversible* binding with diffusion is often captured by having a slow effective diffusion coefficient (pg. 326, [4]). Binding of many antibodies to cells is not truly irreversible, but given the slow internalization, this is effectively irreversible[5]. Finally, the step must immobilize the agent, since enzymatic cleavage in the extracellular space is irreversible but does not immobilize the cleavage products. They are free to continue diffusing in the tissue.

The reaction term can vary in form (e.g. 1^st^ order, saturable, etc.) and is scaled here for 1^st^ order reactions.

Non-dimensionalize

where

And defining the Damkohler number:

where

For antibodies, the Damkohler number is:

**Summary of Three Dimensionless Numbers**

And the characteristic rates are:

Reaction rate = k_rxn_

Diffusion rate =

Permeability rate =

Blood flow rate =

The characteristic concentration is the free (unbound) drug concentration which undergoes reaction, diffusion, and permeability.

**Cellular Uptake**

Blood

Cellular uptake of drugs (e.g. RBC uptake) was ignored in the present simulations. While the cellular uptake in the blood represents a significant pharmacokinetic compartment and is important, for example, in determining volume of distribution[6], the kinetics are generally slower than blood flow. Given a vessel segment of 500 μm and tumor blood flow velocity of 20-400 μm/s, an RBC is only in the vessel segment for ~1-25 seconds. While plasma binding and dissociation can occur on this time scale, generally red blood cell uptake and release occur over longer time scales and could therefore not replenish the plasma. This may be important in cases where vessel flow stops, however (e.g. acute hypoxia). If cell uptake occurred within fractions of a second, this could be accounted for by simply adjusting the fraction bound parameter. Exchange kinetics that occur over the same time scale as blood flow along the length of the vessel would have to be explicitly modeled using numerical simulations.

Tissue

A mechanistic description of cellular kinetics (local binding and metabolism) is a difficult task given the large number of mechanisms that occur for different drugs. Several approaches are possible. First, a large generic framework can be constructed (e.g. >20 rate constants) where most of these rates will be irrelevant (low sensitivity) for any given drug. This complete description would include reversible non-specific binding to extracellular components, reversible specific saturable binding to extracellular components, reversible specific binding to cell surface proteins followed by endocytosis, recycling, and/or degradation, metabolic degradation in the extracellular matrix, reversible passive diffusion across the cell membrane, active transport across the cell membrane (e.g. p-glycoprotein), reversible intracellular binding (non-specific), reversible saturable binding to intracellular components, intracellular metabolism, and loss of metabolic by-products (if they are being measured). This approach is needlessly complicated in most scenarios and makes intuitive understanding more difficult. For example, passive diffusion of antibodies across cell membranes can safely be ignored as can cell surface sticking and endocytic uptake for a small molecule drug that can easily and more rapidly diffuse across the cell membrane.

A second approach is a simplified model with generic rate constants (e.g. reversible step followed by an irreversible step) where these rate constants correspond to different mechanisms for different drugs. For example, the reversible binding of an IgG to cell surface proteins corresponds to the reversible rate constants for antibodies, and the irreversible rate would be the internalization and degradation rate. For lipophilic small molecule drugs, the reversible rate constants could correspond to passive diffusion across the plasma membrane, and the irreversible step would be any metabolism within the cell (e.g. reaction of alkylating agents with DNA). While the abstract mathematical representation may be the same, we have chosen not to use this framework because the model loses its mechanistic interpretation. The ambiguous rate constants correspond to different mechanisms depending on the drug.

Finally, a mechanistic description of the relevant steps can be defined for each drug, albeit the equations may be different for different drugs. We have chosen the third option for several reasons. This results in much simpler cellular kinetics (relative to the first approach), and often only a couple rate constants are needed. These simpler equations are much more amenable to numerical simulation given the stiff nature of the two spatial dimension method of lines method. It also aligns with the reductionist approach to only include the relevant (i.e. high sensitivity) parameters. While the second option of using a generic framework simplifies the math, it loses the mechanistic interpretation of the rate constants. Understanding the mechanism is of central importance in being able to use these simulations to design better drugs and imaging agents. If the rate constant that immobilizes the drug is based on passive diffusion across the membrane, then changing the drug size, charge, and/or lipophilicity will change the distribution. However, if the immobilization is based on cell surface binding kinetics, then engineering a different binding kinetics will be important.

**The local binding and metabolism rate in the Damkohler number is the first effectively irreversible immobilization step.** The three key concepts are immobilization, irreversible, and effectively, discussed in turn. This rate must constitute an immobilization reaction (e.g. cell binding) since otherwise the molecule is still free to diffuse. For example, cleavage of a soluble probe (e.g. an MMP protease sensor) would not immobilize the probe. The reaction must also be irreversible, since rapid binding and dissociation, while it may slow down the diffusion rate, can still allow diffusion and homogenous distribution. Many small molecule drugs reversible and non-specifically bind proteins, which slows the effective diffusion coefficient, but it does not immobilize the drug. The final term, effectively, is seemingly arbitrary, but it defines the relative time scale of the experiment. Many drugs distribute heterogeneously at early times but are homogenous at later times. Over short times, the dissociation of a drug from a target may be negligible, so it is ‘effectively’ irreversible. At later times, the drug may have dissociated and diffused homogenously through the tissue. This complication arises from the fact that drug distribution is a dynamic process, and it is not possible to capture all the information in simple dimensionless groups. Nevertheless, these groups help quickly define the type of behavior a drug displays in tumors without requiring full numerical simulations.

While defining the relevant kinetics for drug uptake is challenging, below are 7 examples to illustrate some of these differences.

Antibodies [7,8,9,10]

The intracellular degraded antibody fragments and label are sometimes simulated in addition to the intact protein. For example, a radiolabel can reside inside the cell long after the antibody is degraded, and the k_loss_ rate constant depends on the residualizing nature of the radioisotope. For ADCC or blocking the dimerization of receptors, simulating degraded fragments is not necessary.

where [Ab] = free antibody, [B] = bound complex, [Ag] = unbound antigen, D = diffusion coefficient, k_on_ = association rate, ε = void fraction, k_off_ = dissocation rate, k_e_ = endocytosis rate, R_s_ = antigen synthesis rate, [I] = intracellular degraded label, and k_loss_ = loss rate of degraded tag from cell.

Endocytic Protease Sensors [11]

where [I]_i_ = inactive protease sensor, D = diffusion coefficient, k_pino_ = pinocytosis rate of the cells, and [A] = activated intracellular protease sensor.

Cisplatin [12,13]

where [C]_ext_ = extracellular concentration of cisplatin (mol/total tumor volume), k_in_ = cell uptake rate, k_out_ = cell loss rate, [C]_int_ = intracellular concentration (mol/total tumor volume), k_rxn_ = DNA reaction rate, k_repair_ = repair rate of DNA damage, and [C]_DNA_ = concentration of DNA adducts. Note that k_in_ = (1-ε)k_1_ from [12,13], and k_out_ = k_1_’. See below for a note on units.

Doxorubicin [14,15,16]

where K_e_ = extracellular exchange coefficient, K_i_ = intracellular exchange coefficient. All concentrations are given in overall tumor concentrations necessitating the void fraction. Although cellular uptake is assumed to be passive, pi-oribtal interactions of the drug at high concentrations reduces uptake, resulting in a different form.

Taxol [17]

For this model, cellular uptake was ignored relative to target binding. The doses were assumed to be low enough that non-specific uptake was negligible.

FDG

Note that no cellular loss of pFDG occurs on the time scale of the experiments due to rapid decay of F-18. For longer time scales, the phosphatase activity would need to be considered. It is also assumed that glucose levels are much higher than FDG levels resulting in linear consumption kinetics.

Oxygen

Note on Units

Care must be taken when converting rate constants from in vitro cell culture experiments to in vivo models. Unless otherwise noted, the above concentrations are in pseudo-homogenous overall tissue volume units (i.e. mol of drug per total tissue volume). For example, the void fraction arises in the binding rate constant for antibodies because diffusing antibody is restricted to the extracellular tissue volume. Therefore, the interstitial concentration (mol of drug per extracellular volume) is higher than the overall concentration.

El-Kareh and Secomb chose to use extracellular and intracellular concentrations for their cisplatin model. For illustration purposes, the pseudo-homogenous equations above are converted back to their extracellular and intracellular concentrations to demonstrate the role of the void fraction:

where

where

The last equation matches equation 4 from [12] expressed in concentrations of interstitial volume (for extracellular) and per cell volume (for intracellular). Since the void fraction has no units, it becomes more difficult to locate errors in the equation, requiring extra caution.

**Parameterization**

| **Parameter** | **Determining Factors** | **References** |
| --- | --- | --- |
| Blood Flow | Tumor blood flow rate  logP, acid/base/neutral molecule (plasma binding) | [18,19,20] |
| Permeability | Vascularization, permeability factors (e.g. VEGF), molecular size | [21,22,23] |
| Diffusion | Void fraction, extracellular matrix composition,  molecular size | [24,25,26] |
| Binding and Metabolism | Highly specific to molecule/target of interest | [7,11] |
| General References |  | Macromolecules [27]  Small molecules [28] |

Supplementary Table 1 – Useful references for parameter estimation

Oxygen

Blood flow rates for all molecules were identical for comparative purposes. A typical blood flow rate for tumors is 0.1 mL/g/min[19]. The bulk blood flow does not completely describe the geometry, so the length of the capillary segment must also be defined. Blood vessel formation is heterogeneous in tumors, but we assume a 500 μm length[29,30].

A large fraction of oxygen in the blood is bound to hemoglobin, and this binding exhibits positive cooperativity[31]. The total oxygen dissolved in the blood is therefore:

where [C]_O_ is the oxygen concentration not bound to hemoglobin, [C]_sat_ = H*20.5x10^-6^ mol/cm^3^ where H is the hematocrit (assumed to be 0.45), [C]_Hb_ = 3.73x10^-8^ mol/cm^3^, and n = 2.66[31]. Given that most plasma binding by small molecules is linear, and the goal is to develop a generic framework, a linearized model of oxygen binding to hemoglobin was used. Under the lower oxygen tensions found in tumors, the 1^st^ order approximation provides a reasonable description, and the fraction bound can be easily extended to small molecule drugs.


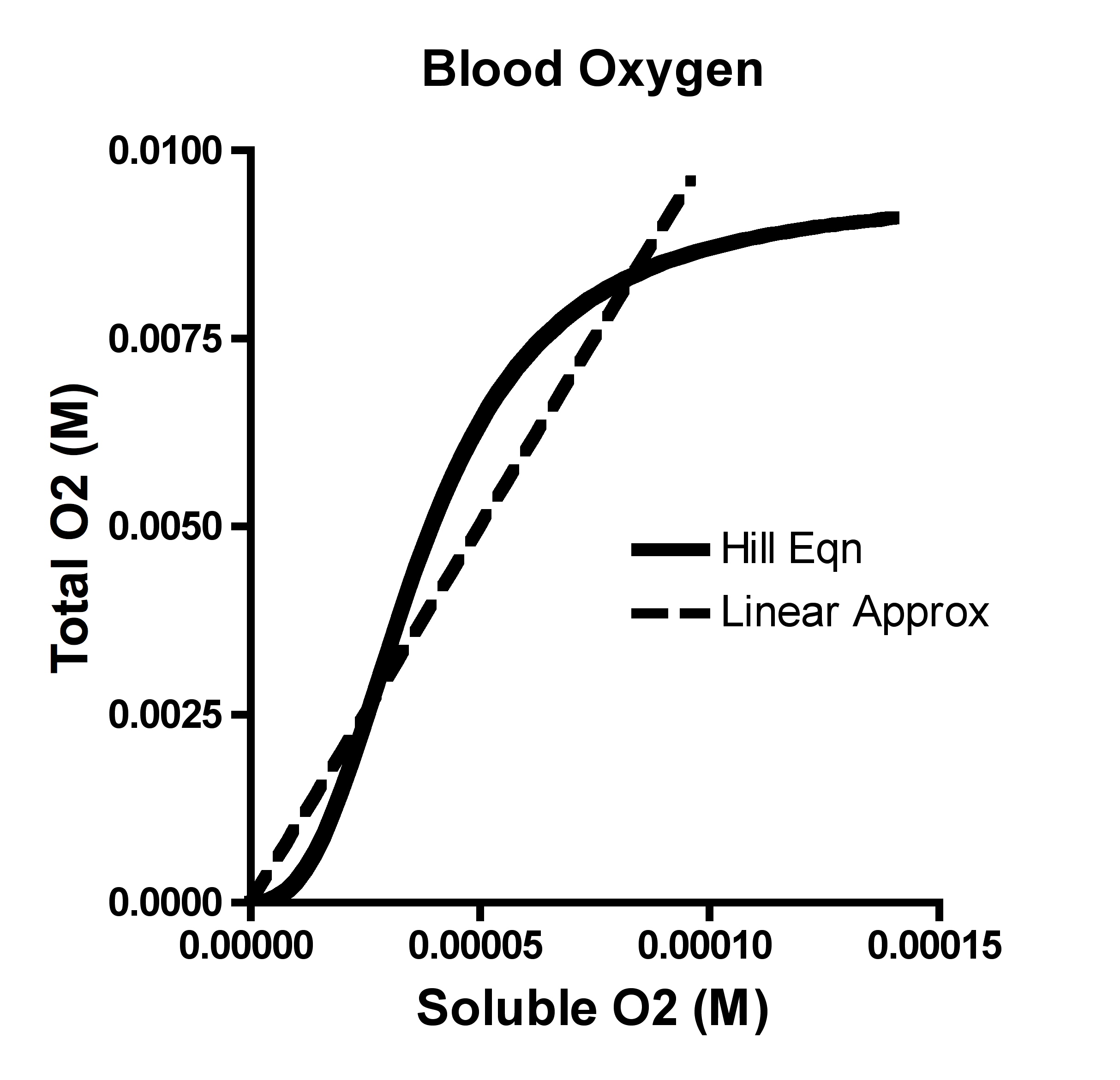


This approximation is not suitable for well-oxygenated tissues due to the divergence of the Hill plot and linear approximation at high oxygen tensions.

For oxygen permeability across the vascular endothelium, the value was based on several assumptions. First, the endothelium is assumed to be 0.6 μm thick, a single diffusion coefficient measured in membranes is used (291 μm^2^/s)[32], and the curvature of the vessel is ignored. The transport rate is then:

= 485 μm/s

Other estimates of permeability are much higher with a thickness of 0.3 μm and diffusion coefficient of 873 μm^2^/s[30], resulting in a larger Biot number and lower concentration drop across the vessel wall.

For the diffusion coefficient in tissue, the value of 1500 μm^2^/s was used[33].

For reaction in tissue, a Michaelis-Menten expression is used. The K_m_ for liver tissue was measured as 2.2 mmHg (~3.0 μM) by Buerke et al.[34] Although the value in brain is much lower, this is similar to the value post-hypoxia in the brain[35]. The equation for consumption is:

For tumor consumption rates, V_max_ in breast and brain tumors ranged from < 2 to 40 μL_O2_/g/min[19]. A value of 8 μL_O2_/g/min will be used for a base case. Using a unit conversion of 1.4276 mg O_2_ per mL_O2_ (i.e. 45 μmol/mL_O2_), this results in 6 μM/s.

Since oxygen is continually supplied in the blood, no systemic clearance was assumed.

FDG

Blood flow rates were identical to those used for oxygen above, and there is no protein binding.

For FDG permeability, Duran et al. report a value of 0.213 μm/s for glucose in normal heart[36]. However, tumor vasculature has an increased permeability, so this provides a lower limit. The hydrodynamic radius of glucose is 4.2 angstroms[37], which using estimates of permeability based on size from Schmidt et al.[27], gives a tumor permeability of 10^-4^ cm/s or 1 μm/s. This is about 5 times what normal tissue is, which generally corresponds to the 3 to 10-fold increased permeability seen with macromolecules.

For diffusion, a coefficient of 500 μm^2^/s [33] is used with a void fraction of 0.44[38]. The diffusion coefficients of oxygen and FDG fall within the range of small molecule solutes in aqueous media[39].

FDG is a tracer dose and competes with glucose in tissues. (This differs from oxygen and antibodies, where there is no competitive species in the simulations.) For Michaelis Menten kinetics of glucose consumption, the rate of glucose consumption is:

Normal human glucose concentrations in the blood are ~100 mg/dL or 1 g/L. The molecular weight of glucose is 180 g/mol, resulting in a blood concentration of ~5.6 mM. For mice, Ferl et al.[40] injected 18.5 MBq, resulting in ~9 MBq/mL. Specific activity is approximated as 10 GBq/μmol[41], so this is an ~1 μM concentration (6,000 fold lower than glucose). For humans, 0.37 to 3.7 GBq is injected, which results in ~0.1 to 1 MBq/mL (from package insert). Assuming FDG behaves identically to glucose, the fraction of FDG is:

The fractional use of FDG will then be:

Assuming the glucose concentration is at steady state, the reaction rate is 1^st^ order:

For lung tumors, Vaupel et al.[19] measured V_max_ of 0.11 μmol/g/min from PET derived data[19]. This results in an estimate of 3.27x10^-4^/s. Nolop et al.[38] measured a rate of 211.4 mL/100g/hr in lung carcinomas, and this gives a similar value of 5.87x10^-4^/s. The rate depends on the individual tissue and can be elevated under hypoxic conditions[42]. These estimates assume this rate is irreversible (i.e. all FDG is phosphorylated inside the cell, and there is no dephosphorylation and loss of the FDG from the cell on the time scales involved).

For clearance, a 1 μM initial concentration is used with 98% alpha decay, k_α_ = 3.2x10^-3^/s, and k_β_ = 3.8x10^-5^/s[43] which gives similar results to Ferl et al.[40]

Doxorubicin

Blood flow parameters were identical to oxygen. Other parameters are in the following table:

| Symbol | Parameter | Value | Reference |
| --- | --- | --- | --- |
| f_free_ | Fraction of unbound drug in plasma | 0.25 | [15] |
| C_0,plasma_ | Initial dose | 25 mg/m^2^  22.7 μM | Typical dose, assuming 1.73 m^2^ body surface and 3.5 L plasma volume. Three clinical studies cited where doses were around 25 mg/m^2^ [14]. |
| MW | Molecular weight | 543.5 g/mol |  |
| P | permeability | 2.8 μm/s | [15] |
| D | Diffusion coefficient | 160 μm^2^/s | [14,44] |
| ε | Void fraction | 0.4 | Small molecule in tumor (extracellular) |
| t_1/2,α_ | Alpha phase plasma clearance | 4.75 min | [14] |
| t_1/2,β_ | Beta phase plasma clearance | 10.23 hr | [45] |
| A | Fraction alpha | 0.999 | [45] |
| V_max_ | Maximum dox exchange rate | 42.9 nM/s | [14] assuming 5x10^8^ cells/mL. Increased the K_i_ by 5-fold to better fit long term clearance (48 hr half life of [45]). Within the equilibrium values of 30-1000 of [14] (value now 300 instead of 60). |
| K_e_ | Extracellular constant | 403 nM |
| K_i_ | Intracellular constant | 63 μM |

Since there is albumin in the interstitial space which doxorubicin can be reversibly, the diffusion coefficient was scaled by this binding. The albumin concentration in the interstitium is ~80% of that found in the plasma[15], so albumin in excess (much higher than drug concentration). From [4], if binding is linear and the fraction of immobilized and free is:

then

and R = 3 for doxorubicin, since 0.75/0.25 is bound/free

The reaction rate includes intracellular and extracellular doxorubicin using the following equations:

Antibodies and Protease Sensors

The same blood flow parameters as oxygen were used, and there is no plasma binding for either of these macromolecules. References for permeability, diffusion, and reaction are given in the table below.

| Agent | Permeability | Diffusion | Reaction | Clearance |
| --- | --- | --- | --- | --- |
| Antibodies | 3x10^-3^ μm/s [46] | 10 μm^2^/s [8]  0.2 | 10^5^/M/s [8]  660 nM | A = 0.43 [7]  k_α_ = 1.5x10^-5^/s  k_β_ = 2.2x10^-6^/s |
| Protease Sensors | 1x10^-3^ μm/s [27] | 10 μm^2^/s [8]  0.2 | 1.1x10^-5^/s [11] | A = 0.57 [11]  k_α_ = 1.95x10^-4^/s  k_β_ = 6.55x10^-6^/s |

The protease sensors are internalized by cells at the rate given above with subsequent loss from an intracellular compartment at a 15 hr half life.

Antibodies undergo saturable binding in the tissue according to the following equations[11]:

**Additional Model Validation**

Oxygen


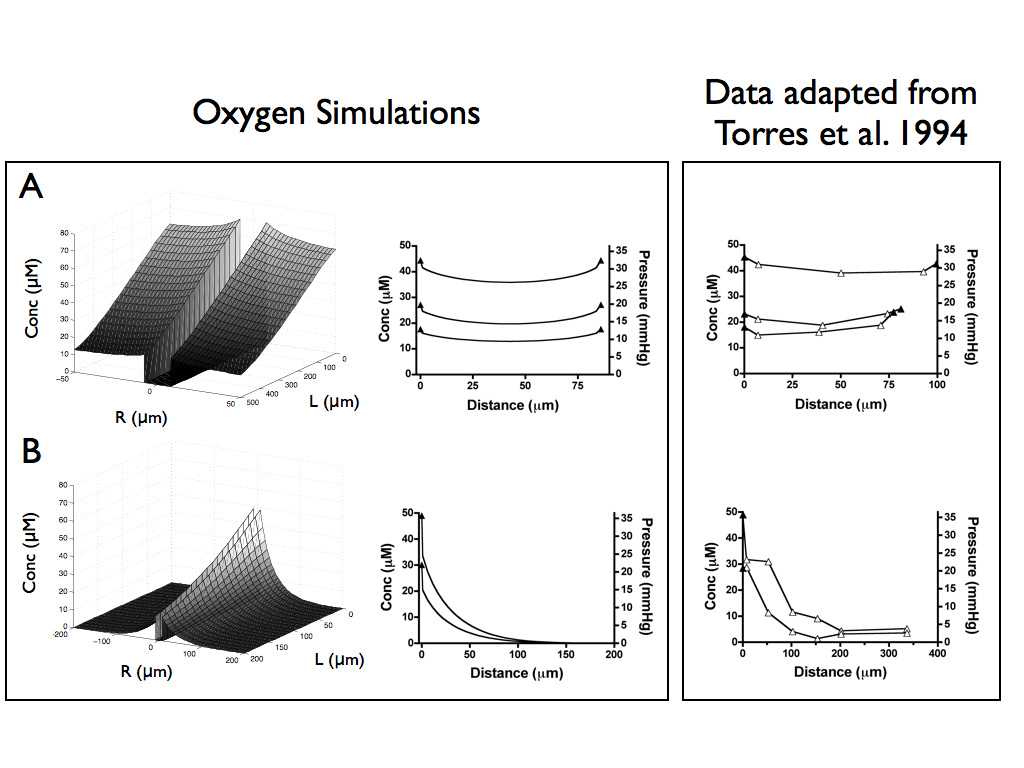


Supplemental Figure 2 – Oxygen profiles from different distances along the radius were plotted with the corresponding plasma concentration with filled triangles. This data is shown beside data adapted from Torres et al.[47] with good agreement. For the experimental data, filled triangles are in the blood while open triangles are in the tissue.

Doxorubicin


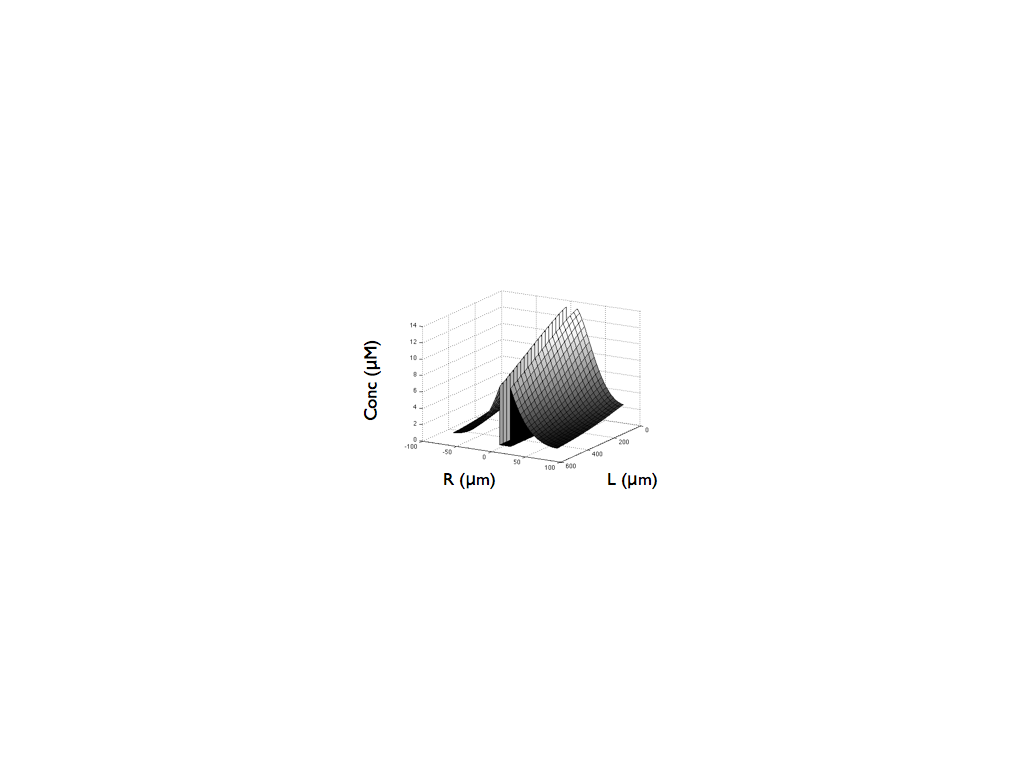


Supplemental Figure 3 – Doxorubicin simulations show both axial and radial gradients. The radial gradients arise from slow tissue penetration and the intracellular accumulation of the DNA binding drug consistent with experimental results[48,49]. The axial gradients are indicative of the blood flow limited uptake.

**Works Cited**

1. Baxter L, Jain RK (1989) Transport of Fluid and Macromolecules in Tumors: 1. Role of Interstitial Pressure and Convection. Microvascular Research 37: 77-104.

2. Baxter LT, Jain RK (1991) Transport of Fluid and Macromolecules in Tumors: 4. A Microscopic Model of the Perivascular Distribution. Microvascular Research 41: 252-272.

3. Coleman PJ, Brashear KM, Askew BC, Hutchinson JH, McVean CA, et al. (2004) Nonpeptide alpha(v)beta(3) antagonists. Part 11: Discovery and preclinical evaluation of potent alpha v beta(3) antagonists for the prevention and treatment of osteoporosis. Journal of Medicinal Chemistry 47: 4829-4837.

4. Crank J (1975) The Mathematics of Diffusion. Oxford: Clarendon Press. 414 p.

5. Mattes MJ, Griffiths G, Diril H, Goldenberg D, Ong G, et al. (1994) Processing of Antibody-Radioisotope Conjugates after Binding to the Surface of Tumor Cells. Cancer 73: 787-793.

6. Hinderling PH (1997) Red blood cells: A neglected compartment in pharmacokinetics and pharmacodynamics. Pharmacological Reviews 49: 279-295.

7. Thurber GM, Zajic SC, Wittrup KD (2007) Theoretic criteria for antibody penetration into solid tumors and micrometastases. J Nucl Med 48: 995-999.

8. Thurber GM, Schmidt MM, Wittrup KD (2008) Antibody tumor penetration: Transport opposed by systemic and antigen-mediated clearance. Advanced Drug Delivery Reviews 60: 1421-1434.

9. Fujimori K, Covell DG, Fletcher JE, Weinstein JN (1990) A modeling analysis of monoclonal antibody percolation through tumors: a binding-site barrier. J Nucl Med 31: 1191-1198.

10. Graff CP, Wittrup KD (2003) Theoretical analysis of antibody targeting of tumor spheroids: importance of dosage for penetration, and affinity for retention. Cancer Res 63: 1288-1296.

11. Thurber G, Figueiredo J, Weissleder R (2009) Multicolor Fluorescent Intravital Live Microscopy (FILM) for Surgical Tumor Resection in a Mouse Xenograft Model. PLoS ONE 4: e8053.

12. El-Kareh AW, Secomb TW (2004) A theoretical model for intraperitoneal delivery of cisplatin and the effect of hyperthermia on drug penetration distance. Neoplasia 6: 117-127.

13. El-Kareh AW, Secomb TW (2003) A mathematical model for cisplatin cellular pharmacodynamics. Neoplasia 5: 161-169.

14. El-Kareh AW, Secomb TW (2000) A mathematical model for comparison of bolus injection, continuous infusion, and liposomal delivery of doxorubicin to tumor cells. Neoplasia 2: 325-338.

15. Eikenberry S (2009) A tumor cord model for Doxorubicin delivery and dose optimization in solid tumors. Theoretical Biology and Medical Modelling 6: 20.

16. Kerr DJ, Kerr AM, Freshney RI, Kaye SB (1986) Comparative Intracellular Uptake of Adriamycin and 4'-Deoxydoxorubicin by Non-Small Cell Lung-Tumor Cells in Culture and Its Relationship To Cell-Survival. Biochemical Pharmacology 35: 2817-2823.

17. Kuh HJ, Jang SH, Wientjes MG, Au JLS (2000) Computational model of intracellular pharmacokinetics of paclitaxel. Journal of Pharmacology and Experimental Therapeutics 293: 761-770.

18. Jain RK (1999) Transport of Molecules, Particles, and Cells in Solid Tumors. Annual Reviews in Biomedical Engineering 01: 241-263.

19. Vaupel P, Kallinowski F, Okunieff P (1989) Blood-flow, oxygen and nutrient supply, and metabolic microenvironment of human-tumors - a review. Cancer Research 49: 6449-6465.

20. van de Waterbeemd H, Testa B (2009) Drug Bioavailability; Mannhold R, Kubinyi H, Folkers G, editors. Morlenbach, Germany: Wiley-VCH.

21. Dreher MR, Liu WG, Michelich CR, Dewhirst MW, Yuan F, et al. (2006) Tumor vascular permeability, accumulation, and penetration of macromolecular drug carriers. Journal of the National Cancer Institute 98: 335-344.

22. Wu NZ, Klitzman B, Rosner G, Needham D, Dewhirst MW (1993) Measurement of Material Extravasation in Microvascular Networks Using Fluorescence Video-Microscopy. Microvascular Research 46: 231-253.

23. Yuan F, Krol A, Tong S (2001) Available Space and Extracellular Transport of Macromolecules: Effects of Pore Size and Connectedness. Annals of Biomedical Engineering 29: 1150-1158.

24. Krol A, Nagaraj S, Dewhirst M, Yuan F (2000) Available volume fraction of macromolecules in tumor tissues. Faseb Journal 14: A167-A167.

25. Brown EB, Boucher Y, Nasser S, Jain RK (2004) Measurement of macromolecular diffusion coefficients in human tumors. Microvasc Res 67: 231-236.

26. Pruijn FB, Patel K, Hay MP, Wilson WR, Hicks KO (2008) Prediction of tumour tissue diffusion coefficients of hypoxia-activated prodrugs from physicochemical parameters. Australian Journal of Chemistry 61: 687-693.

27. Schmidt MM, Wittrup KD (2009) A modeling analysis of the effects of molecular size and binding affinity on tumor targeting. Molecular Cancer Therapeutics 8: 2861.

28. El-Kareh AW, Secomb TW (1997) Theoretical models for drug delivery to solid tumors. Critical Reviews in Biomedical Engineering 25: 503-571.

29. Levitt DG (1972) Capillary-Tissue Exchange Kinetics - Analysis of Krogh Cylinder Model. Journal of Theoretical Biology 34: 103-&.

30. Patton JN, Palmer AF (2006) Numerical simulation of oxygen delivery to muscle tissue in the presence of hemoglobin-based oxygen carriers. Biotechnology Progress 22: 1025-1049.

31. Lightfoot EN (1974) Transport Phenomena and Living Systems. New York: Wiley.

32. Uchida K, Matsuyama K, Tanaka K, Doi K (1992) Diffusion-Coefficient for O2 in Plasma and Mitochondrial-Membranes of Rat Cardiomyocytes. Respiration Physiology 90: 351-362.

33. Kirkpatrick JP, Brizel DM, Dewhirst MW (2003) A mathematical model of tumor oxygen and glucose mass transport and metabolism with complex reaction kinetics. Radiation Research 159: 336-344.

34. Buerk DG, Saidel GM (1978) Local Kinetics of Oxygen-Metabolism in Brain and Liver-Tissues. Microvascular Research 16: 391-405.

35. McGoron AJ, Nair P, Schubert RW (1997) Michaelis-Menten kinetics model of oxygen consumption by rat brain slices following hypoxia. Annals of Biomedical Engineering 25: 565-572.

36. Duran WN, Yudilevich DL (1978) Estimate of Capillary-Permeability Coefficients of Canine Heart to Sodium and Glucose. Microvascular Research 15: 195-205.

37. Schultz SG, Solomon AK (1961) Determination of Effective Hydrodynamic Radii of Small Molecules by Viscometry. Journal of General Physiology 44: 1189-&.

38. Nolop KB, Rhodes CG, Brudin LH, Beaney RP, Krausz T, et al. (1987) Glucose-Utilization In Vivo by Human Pulmonary Neoplasms. Cancer 60: 2682-2689.

39. Blanch HW, Clark DS (1997) Biochemical Engineering: Marcel Dekker. 702 p.

40. Ferl GZ, Zhang XL, Wu HM, Huang SC (2007) Estimation of the F-18-FDG input function in mice by use of dynamic small-animal PET and minimal blood sample data. Journal of Nuclear Medicine 48: 2037-2045.

41. Lemaire C, Damhaut P, Lauricella B, Mosdzianowski C, Morelle JL, et al. (2002) Fast [F-18]FDG synthesis by alkaline hydrolysis on a low polarity solid phase supports. Journal of Labelled Compounds & Radiopharmaceuticals 45: 435-447.

42. Signorelli S, Jennings P, Leonard MO, Pfaller W Differential Effects of Hypoxic Stress in Alveolar Epithelial Cells and Microvascular Endothelial Cells. Cellular Physiology and Biochemistry 25: 135-144.

43. Liu RS, Chou TK, Chang CH, Wu CY, Chang CW, et al. (2009) Biodistribution, pharmacokinetics and PET Imaging of [F-18]FMISO, [F-18] FDG and [F-18]FAc in a sarcoma- and inflammation-bearing mouse model. Nuclear Medicine and Biology 36: 305-312.

44. Weinberg BD, Patel RB, Exner AA, Saidel GA, Gao JM (2007) Modeling doxorubicin transport to improve intratumoral drug delivery to RF ablated tumors. Journal of Controlled Release 124: 11-19.

45. Formelli F, Carsana R, Pollini C (1986) Comparative Pharmacokinetics and Metabolism of Doxorubicin and 4-Demethoxy-4'-O-methyldoxorubicin in Tumor-Bearing Mice. Cancer Chemotherapy and Pharmacology 16: 15-21.

46. Yuan F, Dellian M, Fukumura D, Leunig M, Berk D, et al. (1995) Vascular Permeability in a Human Tumor Xenograft: Molecular Size Dependence and Cutoff Size. Cancer Research 55: 3752-3756.

47. Torres IP, Leunig M, Yuan F, Intaglietta M, Jain RK (1994) Noninvasive Measurement of Microvascular and Interstitial Oxygen Profiles in a Human Tumor in Scid Mice. 91: 2081-2085.

48. Primeau AJ, Rendon A, Hedley D, Lilge L, Tannock IF (2005) The distribution of the anticancer drug doxorubicin in relation to blood vessels in solid tumors. Clinical Cancer Research 11: 8782-8788.

49. Lankelma J, Dekker H, Luque RF, Luykx S, Hoekman K, et al. (1999) Doxorubicin gradients in human breast cancer. Clinical Cancer Research 5: 1703-1707.
